# Age-related loss of chromosome Y is associated with levels of sex hormone binding globulin and clonal hematopoiesis defined by *TET2*, *TP53*, and *CBL* mutations

**DOI:** 10.1126/sciadv.ade9746

**Published:** 2023-04-21

**Authors:** Ahmed A. Z. Dawoud, William J. Tapper, Nicholas C. P. Cross

**Affiliations:** ^1^ School of Medicine, University of Southampton, Southampton, Hampshire SO17 1BJ, UK.; ^2^ Wessex Regional Genetics Laboratory, Salisbury NHS Foundation Trust, Salisbury, Wiltshire SP2 8BJ, UK.

## Abstract

Mosaic loss of the Y-chromosome (LOY) in peripheral blood leukocytes is the most common somatic alteration in men and linked to wide range of malignant and nonmalignant conditions. LOY is associated with age, smoking, and constitutional genetics. Here, we aimed to assess the relationships between LOY, serum biomarkers, and clonal hematopoiesis (CH). LOY in U.K. Biobank was strongly associated with levels of sex hormone binding globulin (SHBG), a key regulator of testosterone bioavailability. Mendelian randomization suggested a causal effect of SHBG on LOY but there was no evidence for an effect of LOY on SHBG. In contrast, age-related CH defined by somatic driver mutations was not associated with SHBG but was associated with LOY at clonal fractions above 30%. *TET2*, *TP53*, and *CBL* mutations were enriched in LOY cases, but *JAK2* V617F was depleted. Our findings thus identify independent relationships between LOY, sex hormone levels, and CH.

## Teaser

Levels of sex hormone binding globulin are linked to age-related loss of the Y chromosome.

## INTRODUCTION

Age-related somatic loss of the Y-chromosome (LOY) in peripheral blood leukocytes is the most prevalent chromosomal alteration in men (*1*). Mosaic LOY, has been identified in as many as 20% of male participants in large population-based cohort studies such as the UK Biobank (UKB; median age = 58), but only 10% of affected individuals (2% of all men) had LOY involving >20% of leukocytes (*2*–*4*). LOY is associated with all-cause mortality, cancer mortality (*1*, *5*, *6*), and a wide range of nonmalignant conditions (*1*, *7*–*9*). LOY is also associated with variation in blood cell counts (*4*, *10*) and has long been recognized as a recurrent clonal cytogenetic finding in hematological malignancies where, in the absence of other changes, it is associated with a good prognosis (*11*). Whether LOY is a direct driver of clonality or merely a passenger event has not been established, but it has been suggested that LOY might be a broad marker of genomic instability across different tissues, or that it may exert its effects by altering immune cell function (*2*, *12*).

Age-related clonal hematopoiesis (CH) is defined in most studies by the finding of specific somatically acquired autosomal chromosomal alterations (*13*, *14*) or a limited range of pathogenic driver mutations (*15*–*20*) in the absence of a hematological neoplasm. The extent to which mosaic LOY, which is much more common than mosaic autosomal alterations, overlaps with CH as defined above has been explored (*18*, *21*) but remains incompletely understood. Both LOY and CH are associated with a wide range of malignant and nonmalignant diseases (*15*, *16*, *22*–*26*). Most prominently, individuals with CH have a significant risk (hazard ratios of 10 to 12.9) of developing a hematological malignancy (*13*, *14*, *16*, *17*), and recent studies showed a lineage-specific risk for mutations in genes associated with myeloid or lymphoid neoplasms (*27*, *28*). LOY with large clone size has been linked to the presence of somatic mutations associated with hematological malignancies and an elevated risk of developing myeloid neoplasia in two recent, small studies of selected cases (*21*, *29*). Although age is the major risk factor for the development of both CH and LOY, it has become clear that there is a substantial overlap in constitutional genetic variation that predisposes to these abnormalities. Broadly, variants in cancer susceptibility genes and genes that are mutated in cancer feature prominently as risk factors for both CH and LOY (*2*, *3*, *20*, *30*, *31*). As for external factors, smoking is associated with both CH and LOY (*1*, *3*, *32*, *33*), indicating that the environment and genetics are important factors.

Serum biochemical profiles are also known to change with age, for example, aging is associated with depletion of sex hormones (*34*), and some of these changes have been linked to common age-related disorders. However, any link between changes in serum markers and LOY or CH remains unexplored. In this study, we aimed therefore to assess the relationships between LOY, CH, and serum biomarkers and investigate the role of constitutional genetics in any associations.

## RESULTS

### The relationship between LOY and biochemistry markers

To investigate the relationship between LOY and serum biomarkers, we used previously published calls of LOY that were generated by using long-range phasing information to analyze allele-specific genotyping intensities of 1239 variants in the pseudo-autosomal region 1 (*2*). We restricted our analysis to the 222,835 males who passed QC, of whom 44,558 (20%) had LOY. Of these, the majority (*n* = 31,952; 72%) had an estimated LOY clonal fraction of <10%. We compared the presence or absence of LOY with 29 biochemistry parameters that were directly measured by UKB, as well as estimated levels of free testosterone (FT; median = 0.21 nM; range = 0.003 to 1.93) and bioavailable testosterone (BAT; median = 5.1 nM; range = 0.09 to 45.68) derived from measurements of sex hormone binding globulin (SHBG), total testosterone (TT), and albumin (*35*).

Univariate comparisons revealed that participants with LOY had higher median levels of alkaline phosphatase, apolipoprotein A, C-reactive protein, cystatin C, glucose, glycated hemoglobin (HbA1c), HDL cholesterol, TT, urea, SHBG, and vitamin D. Lower median levels in participants with LOY were seen for alanine aminotransferase, albumin, apolipoprotein B, aspartate aminotransferase, calcium, cholesterol, γ-glutamyltransferase, insulin growth factor 1, low-density lipoprotein direct, total bilirubin, total protein, triglycerides, urate, and FT (table S1).

**Fig. 1.**
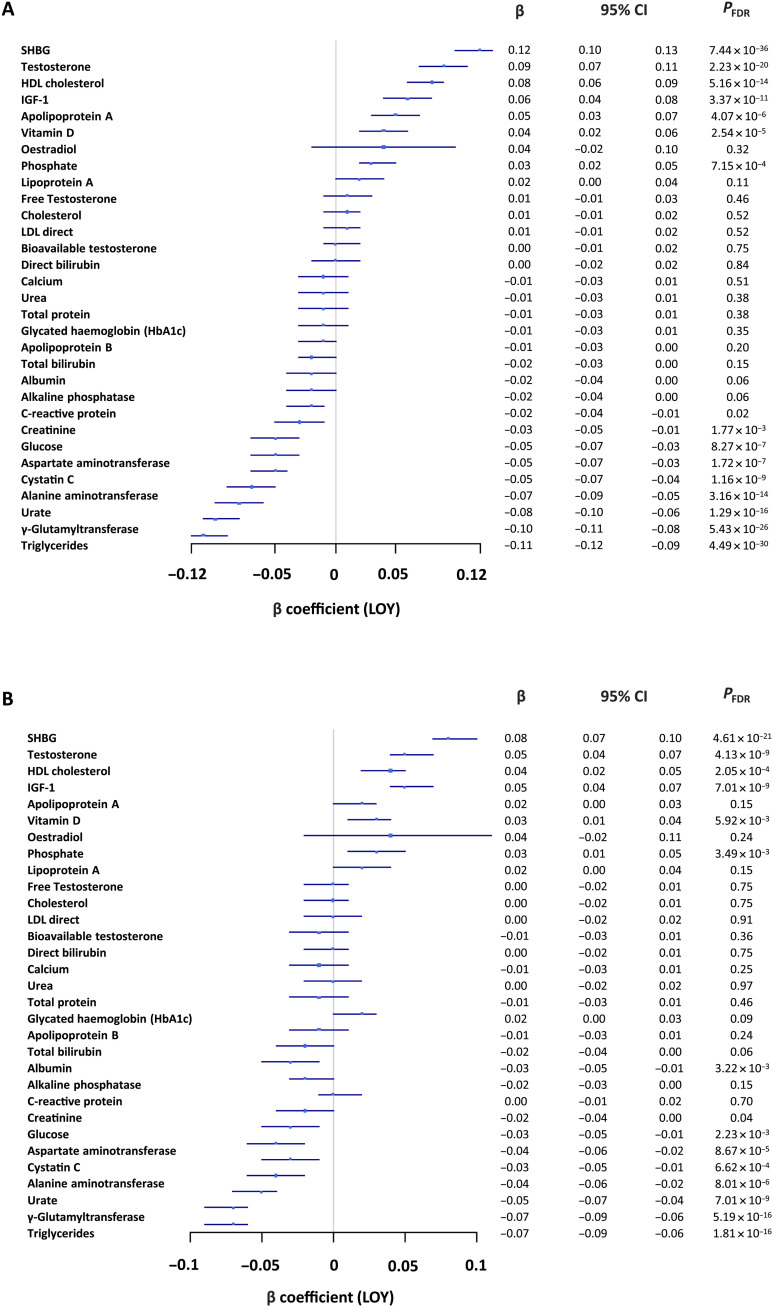
The relationship between LOY and biochemistry markers. The relationship between LOY and each of 31 biomarkers (29 measured and 2 calculated) was tested using multivariable linear regression in R in 222,835 UKB males. (**A**) basic models: each linear model considered age, age squared, smoking status, smoking intensity, 10 PC, and multiple testing; (**B**) sensitivity model: hypertension, insulin-dependent diabetes, non–insulin-dependent diabetes, and BMI were added to each basic model.

On initial multivariate analysis adjusted for age, age squared, smoking status, smoking intensity, the first 10 genetic principal components (10 PC), and using the false discovery rate (FDR) to correct for multiple testing, we found that LOY as a binary predictor was most strongly associated with elevated levels of SHBG [β = 0.12; 95% confidence interval (CI), 0.11 to 0.13; *P* = 7.44 × 10^−36^; Fig. 1]. We therefore focused our subsequent analysis on SHBG, although we note that there are several other potentially important positive and negative associations (Fig. 1).

**Fig. 2.**
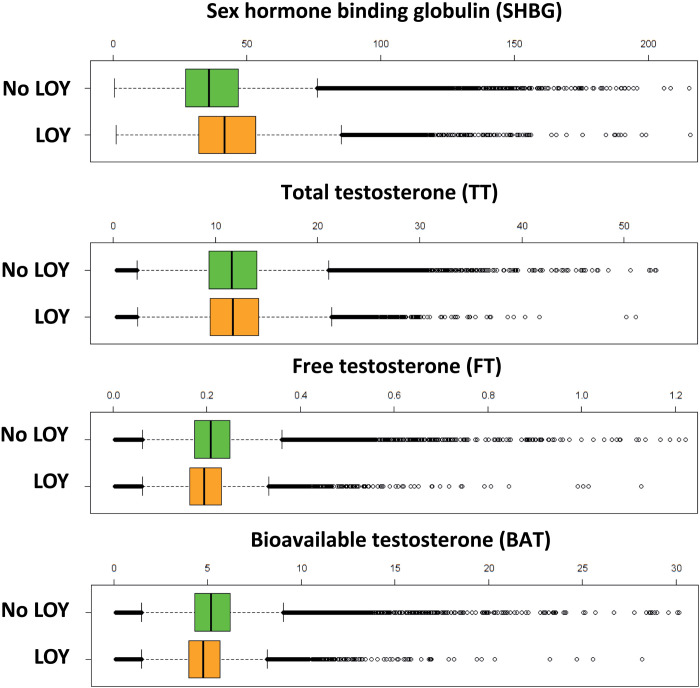
The relationship between LOY and levels of sex hormones. The box plots summarize serum sex hormone measurements in participants without LOY (*n* = 178,277) and with LOY (*n* = 4458). SHBG: median nM = 41.54 versus 35.86, *P* < 0.001; TT: median nM = 11.74 versus 11.58, *P* < 0.001; FT: median nM = 0.19 versus 0.20, *P* < 0.001; BAT: median nM = 4.78 versus 5.18, *P* < 0.001.

SHBG is a glycoprotein that binds to steroids, in particular testosterone, with high affinity (*36*). It is notable therefore that the second strongest positive association was with TT (β = 0.09; 95% CI, 0.07 to 0.11; *P* = 2.23 × 10^−20^). In men, circulating testosterone levels are regulated by SHBG, with on average 58% of TT bound to SHBG, 40% bound to albumin, and 2% present as FT (*37*, *38*). Binding to albumin is weak and so all non–SHBG-bound testosterone is considered as BAT (*39*). Furthermore, aging is associated with a decline in FT and BAT and an increase in SHBG (*40*, *41*). There was no association, however, between LOY and FT (*P* = 0.46) or BAT (*P* = 0.75) (Fig. 1A). Participants with LOY had higher levels of SHBG and TT (SHBG, median nM = 41.54 versus 35.86, *P* < 0.001; TT, median nM = 11.74 versus 11.58, *P* < 0.001; Mann-Whitney *U* tests) but lower levels of FT (median nM = 0.19 versus 0.20, *P* < 0.001) and BAT (median nM = 4.78 versus 5.18, *P* < 0.001). On sensitivity analysis with the addition of hypertension, insulin-dependent diabetes, non–insulin-dependent diabetes, and body mass index (BMI) to the covariates above, LOY retained the positive association with SHBG (β = 0.08; 95% CI, 0.07 to 0.10; *P* = 4.61 × 10^−21^) and TT (β = 0.05; 95% CI, 0.04 to 0.07; *P* = 4.13 × 10^−9^; Fig. 1B and table S2). Our observational results point to a direct relationship between levels of SHBG and LOY that cannot be explained by age, age squared, smoking history or intensity, population stratification, common morbidities, BMI, or free/bioavailable testosterone (Fig. 2).

To assess the possibility that the association between LOY and sex hormones might be due to pleiotropic effects we included a polygenic risk score (PRS) consisting of 156 genome-wide significant single-nucleotide polymorphisms (SNPs) (*2*) as a covariate to account for genetic factors that predispose to LOY. When this score was added as an independent variable in the sensitivity analysis (table S3), LOY retained the positive association with both SHBG (β = 0.08; 95% CI, 0.06 to 0.10; *P* = 8.09 × 10^−18^) and TT (β = 0.06; 95% CI, 0.04 to 0.08; *P* = 1.34 × 10^−9^) but the PRS was not significantly associated with SHBG (*P* = 0.63) or TT (*P* = 0.16). Thus, the association between LOY and SHBG and TT is not explained by established genetic risk factors for LOY.

### The relationship between genetically-defined SHBG and LOY

Published genome-wide association studies (GWAS) have identified multiple genetic determinants of SHBG levels in serum of both men and women (*42*). To understand the relationship between LOY and SHBG, we used an 11 SNP PRS (of which 8 SNPs were associated with SHBG at a level of genome-wide significance and 3 were identified by conditional analysis of the *SHBG* gene) to summarize the genetic variation associated with SHBG and evaluated the score as a predictor of LOY. Since the score was derived from independent cohorts, it represents an unbiased instrument to assess the relationship with LOY in UKB (*43*). We found that genetically predicted SHBG was significantly associated [odds ratio (OR) = 1.02; 95% CI, 1.01 to 1.04; *P* = 5.59 × 10^−5^] with the finding of LOY in UKB.

**Fig. 3.**
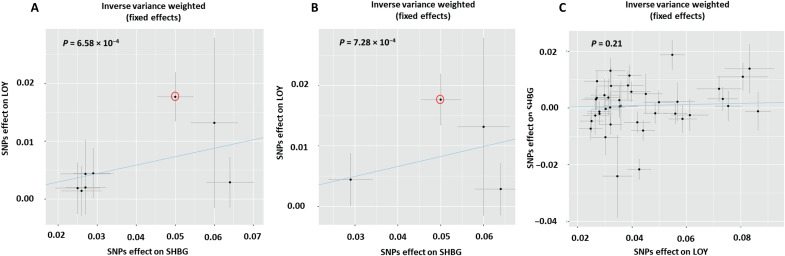
MR using an IVW model to estimate the causal relationship between SHBG and LOY. (**A**) Analysis using eight independent SNPs associated with SHBG (genome-wide significant or derived from conditional analysis) from multiple cohorts of men and women (*42*) used as instrumental variables in BBJ. The IVW test estimated a significant positive effect of SHBG on LOY (*P* = 6.58 × 10^−4^). (**B**) Conservative analysis using a subset of four SNPs associated with SHBG at genome-wide significance in men (*P* = 7.28 × 10^−4^). (**C**) Analysis using 40 independent SNPs associated with LOY in BBJ assessed as instrumental variables in UKB. The IVW test estimated no effect of LOY on SHBG (*P* = 0.21). The line of regression is indicated in blue, and the axes show β coefficients for SNP effects on SHBG and LOY. rs7910927 at 10q21.3 within *JMJD1C* is highlighted with a red circle in (A) and (B) but not in (C) as it was not associated with LOY at a genome-wide significant level in BBJ.

To assess the possibility of a causal relationship between SHBG and LOY, we performed a Mendelian randomization (MR) analysis. Of the 11 SNPs used for the SHBG PRS, 3 were unavailable in Biobank Japan (BBJ) and thus 8 were used to estimate the effect of SHBG on LOY in male BBJ participants (Fig. 3 and tables S4 and S5) (*44*). Using a standard inverse variance weighted (IVW) model with fixed effects, we identified a positive causal relationship (β = 0.15; 95% CI, 0.06 to 0.23; *P* = 6.58 × 10^−4^), with no evidence of heterogeneity (*Q* test, *P* = 0.26). Although not all alternative MR tests identified a significant causal relationship (table S5), effects were consistently seen in the same direction and the nonsignificant Egger intercept (*P* = 0.8; table S5) argues against the possibility of directional horizontal pleiotropy. In a more conservative analysis restricted to the subset of four SNPs that were strongly associated with SHBG in men (*P* < 5 × 10^−8^), the effect of SHBG on LOY was confirmed (IVW, β = 0.17; 95% CI, 0.07 to 0.26; *P* = 7.28 × 10^−4^). Leave-one-out analysis (table S6) found that significance was lost when rs7910927 at 10q21.3 within *JMJD1C* was excluded (initial analysis, *n* = 7 SNPs: β = 0.08; 95% CI, −0.02 to 0.17; *P* = 0.13; and conservative analysis, *n* = 3 SNPs: β = 0.07; 95% CI, −0.04 to 0.19; *P* = 0.22). rs7910927 was associated with LOY in UKB but did not reach genome wide significance (β = −0.02, *P* = 4.59 × 10^−3^) and no association with LOY was seen in the vicinity of *JMJD1C* in BBJ, thus arguing against the possibility of a pleiotropic effect of rs7910927 on both LOY and SHBG. To assess the possibility of a bidirectional effect, we used 40 SNPs with *P* < 5 × 10^−8^ associated with LOY in Japanese men (*44*) to measure their effect on SHBG levels in UKB men (*45*), but no significant relationship was found (β = 0.02; 95% CI, −0.01 to 0.05; *P* = 0.21).

### The effect of gene expression on the relationship between SHBG and LOY

**Fig. 4.**
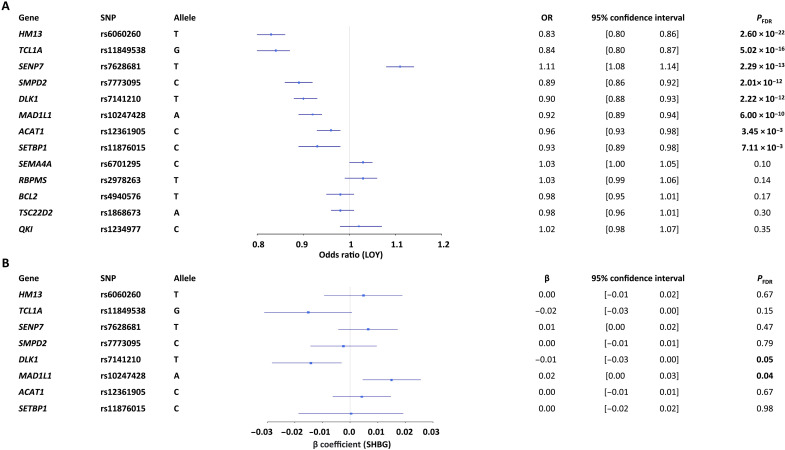
The relationship between the predicted expression of 13 genes and each of LOY and SHBG. eQTL SNPs were used as proxies for gene expression and assessed as predictors for LOY (**A**) and the eight significant SNPs were compared to SHBG levels (**B**) incorporating age, age squared, smoking history and intensity, and 10 PC as covariates.

To explore the hypothesis that germline variants associated with LOY might regulate genes that could modify the association between LOY and SHBG, we focused on 19 genomic regions associated with LOY in UKB (*3*) and replicated a larger UKB dataset and three independent cohorts (*2*). Using the eQTLGene database (*46*), we identified eQTL SNPs to serve as valid proxies for 13 of the 19 regions (*ACAT1*, *BCL2*, *DLK1*, *HM13*, *MAD1L1*, *QKI*, *RBPMS*, *SEMA4A*, *SENP7*, *SENP8*, *SETBP1*, *SMPD2*, *TCL1A*, and *TSC22D2*). We found that 8 of 13 eQTLs were associated with LOY in UKB but only 2 were associated with levels of SHBG, albeit at borderline levels of significance (Fig. 4 and table S7). The eQTL allele associated with increased expression of *MAD1L1* at 7p22.3 was positively associated with SHBG (rs10247428_A, β = 0.02; 95% CI, 0.00 to 0.03; *P* = 0.04), but this was in the opposite direction to its relationship with LOY. The eQTL allele associated with reduced expression of *DLK1*, part of *DLK1-MEG3* imprinted locus at 14q32.2 was negatively associated with SHBG (rs7141210-T, β = −0.01; 95% CI = −0.03 to 0.00; *P* = 0.05; Fig. 4 and table S8) and also negatively associated with LOY. rs7141210 and LOY showed no interactive effects on sex hormones levels, although for SHBG this approached nominal significance (table S9; *P* = 0.07).

### The relationship between LOY, SHBG, and other forms of CH

**Fig. 5.**
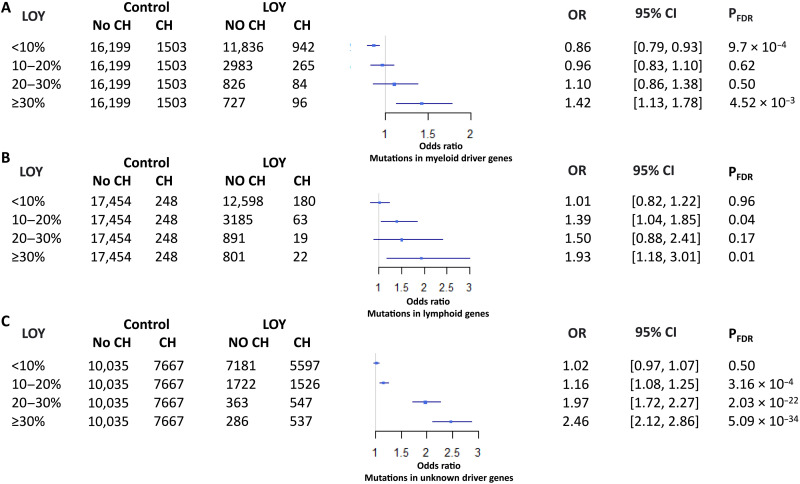
The relationship between LOY and CH. LOY was stratified according to the clonal size and the proportion of participants with CH within each group was compared with controls. (**A**) myeloid CH, (**B**) lymphoid CH, and (**C**) unknown driver CH.

To understand the impact of somatic mutations on the relationship between SHBG, testosterone, and LOY, we assessed the relationship between somatically acquired single-nucleotide variants (SNVs), sex hormones, and LOY. Whole exome sequence (WES) data were available for 17,759 participants with LOY, of whom 28% (*n* = 4981) were estimated to have an LOY clone size ≥10% of leukocytes. For comparison, we randomly selected UKB age-matched male controls (*n* = 17,702) who were negative for LOY. We identified recurrent somatic SNVs in driver genes associated with myeloid CH and lymphoid CH (table S10) plus likely somatic SNVs in other genes. This latter group is expected to be mostly passenger mutations that do not confer a selective advantage but indicate clonality in the absence of known driver mutations, a situation we refer to as “unknown driver CH.” Overall, the frequency of each CH subtype (myeloid, lymphoid, and unknown driver) was similar between cases with LOY and controls. Notable differences emerged, however, when LOY was stratified by clone size. All SNV-associated CH (myeloid plus lymphoid plus unknown driver) was significantly associated with LOY in ≥10% of cells with a clear increase in the strength of the association with increasing LOY clone size (10 to 20% LOY, OR = 1.17, *P* = 1.81 × 10^−4^; 20 to 30% LOY, OR = 2.20, *P* = 4.25 × 10^−27^; ≥30% LOY, OR = 3.43, *P* = 2.42 × 10^−52^; table S11). Similar results were seen for unknown driver CH considered alone (10 to 20% LOY, OR = 1.16, *P* = 3.16 × 10^−4^; 20 to 30% LOY, OR = 1.97, *P* = 2.03 × 10^−22^; ≥30% LOY, OR = 2.46, *P* = 5.09 × 10^−34^). Of note, LOY clone size was also related to age. The mean ages of cases with LOY <10%, 10 to 20%, and ≥30% were 61.7, 64.2, and 65.4, respectively (<10% versus 10 to 20%, *P* = 1.03 × 10^−10^; 10 to 20 versus ≥30%, *P* = 7.4 × 10^−8^) but the associations between CH and LOY clone size remained significant when adjusting for age, age squared, smoking history and intensity, and 10 PC (table S12). By contrast, both myeloid CH (OR = 1.42, *P* = 4.52 × 10^−3^) and lymphoid CH (OR = 1.93, *P* = 0.01) were significantly associated with LOY in ≥30% of cells but not LOY of smaller clone size (Fig. 5). Our findings thus confirm and extend a previously reported association between LOY and unknown driver CH (*17*). Notably, however, none of the three CH SNV–associated subtypes was associated with SHBG or the three measures of testosterone, indicating no effect of driver mutations on the relationship between LOY and sex hormones (table S13).

To understand the relationship between CH and LOY in more detail, we assessed the association between somatic mutations in specific driver genes in participants with LOY in ≥30% cells (high level LOY; *n* = 823) compared to LOY free controls (*n* = 17,702). Focusing on genes (*n* = 9) that had driver mutations in ≥3 cases (table S14), *TET2* was the most significantly enriched mutated gene in LOY cases (4% versus 1.5% in controls, OR = 2.64, *P* = 9.58 × 10^−5^) with *TP53* (OR = 6.96, *P* = 7.62 × 10^−3^) and *CBL* (OR = 7.43, *P* = 0.04) mutations also showing a significant enrichment (Table 1). These relationships remained significant after excluding cases with a diagnosis of cancer at or before study recruitment (*n* = 2998; table S15). We found no significant association between these three genes and SHBG or TT, but lower levels of FT and BAT were noted in men with *TP53* mutations (table S16). The other 6 genes, including *DNMT3A* and *ASXL1*, showed no enrichment in high-level LOY cases and the prevalence of *DNMT3A* mutations was lower in cases with high-level LOY compared to controls, although the difference was not significant (3.8% versus 2.4%; *P* = 0.11). To explore the possibility that some somatic abnormalities might be negatively associated with LOY, and thus potentially missed by focusing on high-level LOY and driver mutations in ≥3 cases, we compared mutational status with LOY at any level. As shown on table S17, *JAK2* V617F but no other gene/SNV was negatively associated with LOY (OR = 0.39; 95% CI, 0.22 to 0.66, *P*_FDR_ = 6.78 × 10^−3^).

**Table 1.** The relationship between LOY with clone size ≥30% and driver mutations on the gene level. Note that other variants associated with CH such as *JAK2* V617F do not appear on this table as they were not mutated in ≥3 males with LOY in ≥30% of cells.

Driver mutations	Control		LOY		OR	95% CIs		*P*_FDR_
	No CH	CH	No CH	CH				
*TET2*	16,199	253	727	30	2.64	1.73	3.90	9.58 × 10^−5^
*TP53*	16,199	16	727	5	6.96	1.99	19.95	7.62 × 10^−3^
*CBL*	16,199	9	727	3	7.43	1.29	29.84	0.04
*NF1*	16,199	15	727	3	4.46	0.83	15.80	0.09
*DNMT3A*	16,199	637	727	18	0.63	0.37	1.01	0.11
*SF3B1*	16,199	36	727	4	2.48	0.64	6.93	0.14
*STAG2*	16,199	113	727	8	1.58	0.66	3.23	0.33
*SRSF2*	16,199	40	727	3	1.67	0.33	5.27	0.49
*ASXL1*	16,199	167	727	7	0.93	0.37	1.98	1.00

**Fig. 6.**
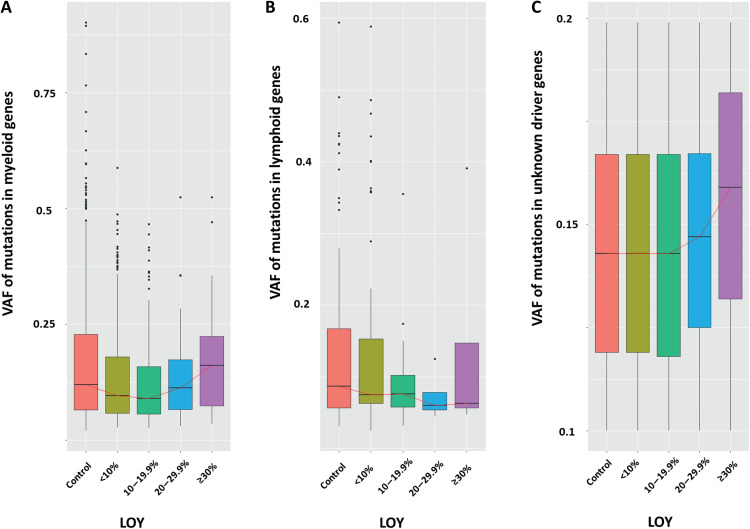
The relationship between LOY clonal size and CH VAFs. Boxplots summarizing the distribution of VAFs of somatic mutations in controls and cases with LOY broken down by clone size. (**A**) myeloid CH, (**B**) lymphoid CH, and (**C**) unknown driver CH. The red lines connect median values.

The possibility that LOY and CH as defined by SNVs might coexist in the same clone was assessed by analyzing the relationship between LOY B-allele frequencies (BAFs) and the variant allele frequencies (VAFs) of driver mutations. Figure 6 shows a summary of the results at different ranges of LOY. Myeloid CH VAFs were associated with BAF levels in samples with LOY ≥10% (β = 0.13; 95% CI, 0.05 to 0.20; *P* = 4.89 × 10^−3^). Similar results were seen for unknown driver CH (β = 0.18; 95% CI, 0.10 to 0.26; *P* = 4.67 × 10^−5^), which by definition was restricted to VAFs of 0.1 to 0.2 (table S18).

## DISCUSSION

Age-related mosaic LOY in peripheral blood leukocytes is known to be influenced by both genetic and environmental factors. We have found that LOY is also strongly associated with multiple serum biomarkers, most notably levels of SHBG, and that this association is independent of known confounders (age, age squared, smoking history, common comorbidities, BMI, and 10 PC). Furthermore, we found genetic evidence to support the hypothesis that SHBG levels are causally linked to LOY, but no evidence that LOY has any effect on SHBG. SHBG regulates the level of circulating testosterone and, although we found that both FT and BAT were lower in men with LOY compared to those without LOY, there was no significant relationship between LOY and either FT or BAT on multivariate analysis. This finding is inconsistent with the free-hormone hypothesis, which proposes that only the unbound fraction of testosterone is biologically active in target tissues (*47*) and instead suggests that other pathways may be involved. The mechanism by which SHBG promotes LOY is unclear but from a genetic perspective, the effect is not explained by variation at *SHBG* alone. Other loci are involved, in particular *JMJD1C*, which encodes a histone demethylase previously linked to SHBG levels (*42*).

To understand the influence of genetic factors on the relationship between SHBG and LOY in more detail, we focused on genetically predicted expression of genes linked to the development of LOY. We identified one eQTL (rs7141210-T, associated with reduced expression of *DLK1*) that was negatively associated with both LOY and SHBG. *DLK1* encodes a delta-like noncanonical notch ligand but this gene is part of the large and complex *DLK1-MEG3* imprinted cluster of genes and noncoding RNAs. The methylated paternally derived chromosome expresses the protein-coding genes *DLK1*, *RTL1*, and *DIO3*, while the nonmethylated maternally derived chromosome expresses the noncoding genes *MEG3*, *MEG8*, *asRTL1*, multiple miRNAs, and lncRNAs (*48*). Constitutional uniparental disomy (UPD) at 14q32 is associated with the developmental disorders Temple syndrome (maternal UPD) and Kagami-Ogata syndrome (paternal UPD), whereas somatically acquired paternal UPD is associated with CH and myeloid malignancies (*49*). Genome-wide significant signals have been identified near *DLK1* in association with CH defined by acquired 14q UPD (*50*) and somatic driver mutations (*51*), as well as LOY (*2*, *3*, *44*). Collectively, these findings suggest that the impact of rs7141210-T on the relationship between SHBG, CH, and LOY merits further investigation.

CH is typically defined by pathogenic driver mutations associated with hematological malignancies, most commonly in the epigenetic regulators *DNMT3A*, *TET2*, and *ASXL1*, which collectively account for 90% of CH cases (*15*–*20*). Broad screens by WES or whole-genome sequencing have revealed that clonality in the absence of known driver mutations (unknown driver CH) is even more prevalent than CH with driver mutations (*18*). In this study, we have defined the relationship between CH and LOY. LOY in ≥30% of cells was associated with both myeloid and lymphoid CH, with 14% of affected individuals having one or more somatic driver mutations compared to 10% of controls (*P* = 2.92 × 10^−4^; table S11). At the level of individual genes, the most notable finding was that mutated *TET2* was associated with LOY but not *DNMT3A* or *ASXL1*. Our findings are consistent with the notion that CH with *TET2* mutations is different from CH with *DNMT3A* or *ASXL1* mutations as indicated by some clinical studies, for example, CH with *TET2* mutations has been linked to chronic obstructive pulmonary diseases (*52*) but not CH with *DNMT3A* mutations.

With regard to the relationship between LOY and CH, our data confirm and extend previous observations (*17*, *18*). As detailed in table S11, unknown driver CH was seen in 65% (537 of 823) of cases with high level (≥30% of cells) LOY compared to just 43% (7667 of 17,702) of controls (*P* = 5.09 × 10^−34^). Overall, 80% (655 of 823) of cases with high-level LOY had mutational evidence of clonality (i.e., CH defined as myeloid, lymphoid, or unknown), with LOY in ≥10% of cells associated with unknown driver CH. Our findings thus provide statistical evidence that LOY ≥10% coexists with CH. We found that the degree of LOY was associated with the VAF of the somatic variants used to define CH (*P* = 3.72 × 10^−12^); however, this finding does not clarify whether LOY might be a direct driver of clonality, as has been postulated recently (*52*), or whether LOY is simply a passenger event associated with as yet uncharacterized factors that promote clonality. For our study, however, neither overall CH nor any CH subtype was associated with SHBG or measures of testosterone (table S13).

In summary, we conclude that (i) SHBG is associated with LOY, but this relationship cannot be explained by the free-hormone hypothesis; (ii) SHBG has a likely causal effect on LOY, but LOY has no effect on SHBG; and (iii) CH does not explain the relationship between LOY and SHBG.

## MATERIALS AND METHODS

### Study cohort

UKB is a large prospective cohort described in detail elsewhere (*53*), involving approximately 500,000 individuals aged between 40 and 69 years at recruitment. Genome-wide SNP data derived from peripheral blood leucocytes were available for most participants and WES data for 200,631 participants at the time of analysis. All participants provided informed consent according to the Declaration of Helsinki, and UKB received ethical approval from the Northwest Multi-center Research Ethics Committee (REC reference 11/NW/0382). The current study was conducted under approved UKB application number 35273.

### Mosaic loss of the Y chromosome

**Fig. 7.**
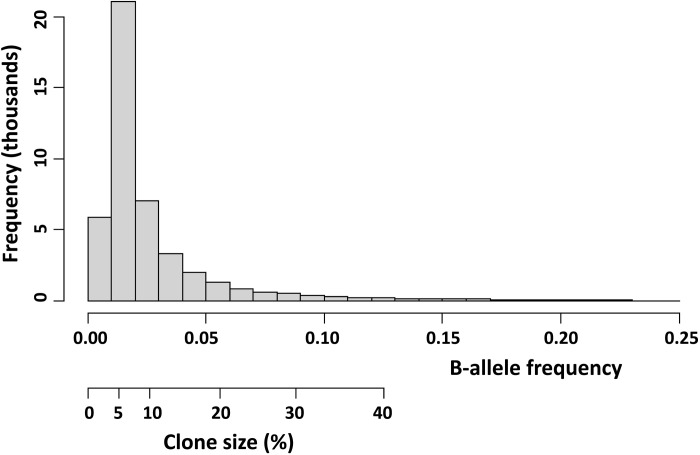
The distribution of BAFs and estimated clone sizes for the 44,558 detected LOY events.

A previous study used SNP data to identify males with mosaic LOY (*n* = 44,588; 20% of evaluable males) using a method which compared allelic intensities for statistically phased haplotypes of the pseudo-autosomal region 1 (PAR1) (*2*). This method for detecting LOY was considered to be less error prone than those based on the median genotyping intensity over the nonpseudoautosomal region of the Y chromosome and was able to detect mosaicism with a clonal fraction down to 1% (*2*), but the majority of events are detected at cell fractions >5% (Fig. 7). The spectrum of LOY was categorized according to clonal fraction by considering the median change of BAF in PAR1; specifically, BAFs of 0.026, 0.056, and 0.088 were previously shown to correspond with clonal fractions of 10, 20, and 30%, respectively (*2*).

### Biochemistry markers and sex hormones

Measurements of 29 biochemistry markers were available from serum samples collected on recruitment to UKB (*53*). Mass action equations were used to calculate FT and BAT from measurements of SHBG, TT, and albumin as described (*54*). Further details regarding the biochemical assay methods and external quality assurance are available at https://biobank.ndph.ox.ac.uk/showcase/showcase/docs/serum_biochemistry.pdf.

### The relationship between biochemistry markers and LOY

We focused on the subset of male UKB participants who were evaluable for LOY assessment (*n* = 222,835) (*2*). WES data were available for 40% of these individuals (*n* = 89,811). The relationship between LOY and each of 31 biomarkers (29 measured and 2 calculated) was tested using multivariable linear regression in R. Continuous measures for each sex hormone were transformed into a normal distribution using inverse normal rank transformation and used as the dependent variable. For the basic model, the independent variables were LOY as a binary predictor, age, age squared, smoking history (never, previous, and current), smoking intensity defined by average number of pack-years smoked by an individual over their adult lifetime (Data-Field 20162) (*55*), and 10 PC. For sensitivity analysis, we added more variables to the basic model, specifically binary variables derived from first occurrences of primary hypertension (ICD10:I10), insulin-dependent diabetes (E10), and non–insulin-dependent diabetes (E11), plus BMI as a continuous variable. Effect sizes were reported as β coefficients (β) with 95% CI. *P* values were adjusted for 31 tests using the FDR method. The average measure of serum biomarkers in participants with LOY were compared to those in LOY free controls using Mann-Whitney *U* tests.

### PRS for LOY in UKB

One-hundred fifty-six genome-wide significant variants were associated with LOY by a previous GWAS (*2*). A PRS was calculated as the sum of number of risk alleles per SNP weighted by their corresponding genetic effect size using the score function in Plink V1.9 and transformed into a normal distribution using inverse normal rank transformation (*56*). We added the PRS to the sensitivity analysis of each serum biomarker. Effect sizes for both LOY and LOY score were reported as β with 95% CI (table S3).

### PRS for SHBG in UKB

Thirteen genetic variants were associated with circulating SHBG levels by a previous two-stage GWAS (*42*) of both men and women from multiple cohorts that did not include UKB or BBJ. The 13 variants included 10 that achieved genome-wide significance plus 3 independent cis variants that were identified by conditional analysis of the *SHBG* gene. After excluding one SNP with heterogeneity toward females (*P* = 0.02, rs440837) and a second SNP on the X-chromosome (rs1573036), 11 SNPs (rs12150660, rs1641537, rs1625895, rs6258, rs17496332, rs2411984, rs293428, rs780093, rs7910927, rs8023580, and rs4149056) were used to calculate an SHBG PRS as described above. A multivariable logistic regression model adjusted for age, age squared, smoking history and intensity, and 10 PC was used to assess the relationship between LOY status (binary and dependent) and the SHBG PRS in UKB.

### Mendelian randomization

MR was used to assess the possibility of a causal relationship between SHBG and LOY using germline SNPs associated with circulating SHBG (*42*) as instrumental variables for LOY in BBJ, following the STROBE guidelines (*57*). Of the 11 SNPs described above for the SHBG PRS, three (rs12150660, rs6258, and rs2411984) were unavailable in BBJ. The remaining eight SNPs (of which six were genome-wide significant for SHBG and two were cis variants identified by conditional analysis of the *SHBG* gene) were thus used as instrumental variables without considering estimated genetic effect sizes for LOY in 95,380 BBJ men (*44*). The analysis was performed using the TwoSamplesMR package (*58*), with the IVW (fixed effects) model considered as the standard method. Other models were used for sensitivity analysis, and the MR-Egger model as a method to account for horizontal pleiotropy. To explore the effect of SNP selection, we repeated the MR analysis using a genome-wide significance threshold specifically in men (*P* < 5 × 10^−8^) to select a subset of four SNPs (rs1641537, rs1625895, rs293428, and rs7910927) associated with SHBG (*42*). We also performed a leave-one-out analysis to evaluate the effect of each SNP on the analysis. To assess the effect of LOY on SHBG levels, we examined SNPs (*n* = 50) previously associated with LOY (*44*) in BBJ. Ten SNPs were excluded as genotypes were unavailable and/or noninformative in UKB. The remaining 40 SNPs were used as instrumental variables without considering estimated genetic effect sizes for SHBG in UKB men.

### Identification of CH

A propensity score matching method in R (*59*) was used to select one control per case and to match for age in comparison to participants with LOY. Somatic mutations were called in LOY samples and matched controls using GATK (version 4.1.9) and Mutect2 (*60*), to process individual CRAM files in the tumor-only mode. Following best practice guidelines (https://gatk.broadinstitute.org/hc/en-us/articles/360035531132), a Panel Of Normal (version 23 August 2017) from the Broad Institute that were generated using Mutect2 on samples from the 1000 genomes project to identify recurrent artifacts, and germline variants from gnomAD were used to remove artifacts and germline variants. To identify putative somatic driver mutations, the analysis was restricted to rare variants with a minor allele frequency (MAF) <0.01 in gnomAD and a minimum number of reads supporting the mutated allele: three reads for point mutations and six reads for indels. Other methodological details for calling somatic variants are described in detail in our previous publication (*24*). Variants that satisfied either of the following two criteria were selected: first, recurrent driver mutations as defined in our previous study (*24*); and second, singleton variants that passed all Mutect2 filters with VAF between 0.1 and 0.2 (*18*). Driver mutations were classified into myeloid or lymphoid according to a published list of genes associated with myeloid neoplasms (*n* = 76) (*15*) and genes associated with acute lymphoblastic leukemia or chronic lymphocytic leukemia in the Cancer Gene Census (*61*), respectively (table S1). Participants with variants in myeloid genes were considered as having myeloid CH (*n* = 2890), and those with variants in lymphoid genes as having lymphoid CH (*n* = 532). Cases with mutations in genes involved in both myeloid and lymphoid neoplasms were considered as myeloid CH. To identify participants with evidence of clonality in the absence of pathogenic mutations in known driver genes, we also identified variants in all coding genes that were not defined as myeloid or lymphoid. Participants with singleton variants in any gene with VAF range between 0.1 and 0.2 not defined as myeloid or lymphoid were considered as having unknown driver CH (*n* = 15,874).

### The relationship between LOY and CH

The association between LOY and all variants, driver variants, myeloid CH, lymphoid CH, and unknown driver CH was tested using logistic regression in R. We further assessed the relationship with LOY clone size categories (<10%, 10 to 20%, 20 to 0%, and ≥30%) using Fisher’s exact tests. Age was compared to LOY clone size using the Wilcoxon signed-rank test. The strength of the association was reported as OR with 95% CI. *P* values were adjusted for 20 tests using the FDR method. To test the relationship at the individual driver gene level, we initially focused on the nine genes mutated in ≥3 males and cases with LOY in ≥30% of cells (Table 1) and then on genes mutated in ≥10 cases + controls and cases with LOY at any level (table S17). Self-reported cancer diagnosed by doctor (UKB: Data-Field 2453) was used to identify participants with prevalent cancer (table S15).

### Assessment of the coexistence of LOY and CH

The coexistence of CH and LOY was tested by assessing the relationship between the BAF for LOY and VAF of driver mutations. For cases with two or more mutations, the highest VAF was used. To avoid excess CH with VAF between 0.1 and 0.2, the analysis for myeloid and lymphoid CH was restricted to VAFs detected for recurrent driver mutations as defined in our previous study (*24*). We assessed the relationship with LOY in ≤10 and >10% of cells (table S18), taking age, age squared, smoking intensity and history, and 10 PC into account. The strength of the association was reported as β coefficient with 95% CI. *P* values were adjusted for six tests using the FDR method.

### The relationship between CH and sex hormones

The association between CH and sex-hormone levels was tested using linear regression in R, with normally transformed sex hormone as the independent variable. The dependent variables were driver mutation state as a binary predictor, age, age squared, smoking status and intensity, and 10 PC. The association was reported as β coefficient with 95% CI. *P* values were adjusted for four tests using the FDR method.

### Expression quantitative trait analyses

To examine the relationship between 19 SNPs associated with LOY (*3*) and SHBG levels, we used the eQTLGen database (*54*) to identify proxies associated with gene expression. Proxies were filtered for cis-eQTLs within a distance <1 Mb and FDR < 0.05. The SNP with the smallest FDR value and no other genes showing a stronger association were selected as proxies to minimize potential horizontal pleiotropy. The analysis was restricted to directly genotyped SNPs with MAF > 0.05 in UKB. The 19 SNPs were associated with 27 genes by position, biological function, expression, or nonsynonymous variants in the gene (*3*). Our analysis was restricted to 13 of these genes for which an expression proxy was identified.

### Assessment of the interaction between eQTL, LOY, and SHBG levels

The most significant eQTL SNP for each gene was encoded according to the risk genotype (0, 1, and 2) in an additive model. The following statistical tests were applied and adjusted for age, age squared, smoking history and intensity, 10 PC, and multiple tests by the FDR method. First, the relationship between each eQTL and LOY was assessed using logistic regression in R where LOY (binary) was the dependent variable and eQTL was the independent variable. Second, the relationship between eQTLs and SHBG levels was assessed using linear regression in R where SHBG (continuous) was taken as the dependent variable and transformed into a normal distribution using rank transformation, and eQTLs were the independent variable. Last, if an eQTL was significantly associated with both LOY and SHBG, then the interaction effect of the eQTL and LOY on SHBG and other sex hormones was assessed by linear regression in R. Inverse normal rank-transformed sex hormone was considered as a continuous dependent variable and each of eQTL, LOY, and eQTL x LOY (interaction effect) as the independent variable.

## Notes

View/request a protocol for this paper from *Bio-protocol*.
