# Supplementary material for: Age-related loss of chromosome Y is associated with levels of sex hormone binding globulin and clonal hematopoiesis defined by TET2, TP53, and CBL mutations
Source: Sci Adv. 2023 Apr 21;9(16):eade9746. doi: 10.1126/sciadv.ade9746 (PMC10121166; doi:10.1126/sciadv.ade9746)
Supplement: Supplementary file 1 — Legends for tables S1 to S18 [file sciadv.ade9746_sm.pdf]

Science Advances
AAAS

Supplementary Materials for

Age-related loss of chromosome Y is associated with levels of sex hormone binding globulin and clonal hematopoiesis defined by *TET2*, *TP53*, and *CBL* mutations

Ahmed A. Z. Dawoud *et al.*

Corresponding author: Nicholas C. P. Cross, ncpc@soton.ac.uk

Sci. Adv. **9**, eade9746 (2023)
DOI: 10.1126/sciadv.ade9746

The PDF file includes:

Legends for tables S1 to S18

Other Supplementary Material for this manuscript includes the following:

Tables S1 to S18

Table S1: Univariate analysis of 31 biochemistry markers.

Table S2: Linear regression results of LOY against 31 biochemistry markers using main model and sensitivity models.

Table S3: Linear regression results of LOY and LOY polygenic risk score (PRS) against 31 biochemistry markers using the full sensitivity models.

Table S4: Mendelian randomization using SNPs associated with SHBG as instrumental variables to assess the causal effect of SHBG on LOY

Table S5: Results of Mendelian Randomization analyses of SHBG levels and LOY

Table S6: Leave-one-out analysis

Table S7: The relationship between eQTLs and LOY

Table S8: The relationship between eQTLs and SHBG

Table S9: The effect of rs7141210-T on the relationship between LOY and sex hormones

Table S10: Genes used to define myeloid CH and lymphoid CH

Table S11: The relationship between LOY and driver mutations

Table S12: The relationship between LOY and driver mutations estimated by binomial logistic regression

Table S13: The relationship between CH and sex hormones

Table S14: Numbers of driver somatic mutations in controls and cases with >=30% LOY

Table S15: The relationship between LOY and driver mutations on gene level excluding men with diagnosed with prevalent cancer (n=2998)

Table S16: The relationship between sex hormones and the three genes *TET2*, *TP53* and CBL

Table S17: The relationship between LOY and CH at the driver gene level

Table S18: The relationship between driver mutations and LOY
